# NETWORK ANALYSIS OF PHYSICAL SYMPTOMS OF OLDER ADULTS WITH COVID-19

**DOI:** 10.1093/geroni/igad104.2266

**Published:** 2023-12-21

## Abstract

**Background:**

COVID-19 can affect multiple organ systems in older adults, making it challenging to manage symptoms that involve different parts of the body. The objectives of our study were to generate symptom networks of COVID-19 physical symptoms in older adults and to explore the core symptoms and the relationships between complex symptoms from a mechanism-based perspective.

**Methods:**

Data from 470 older adults (≥65 yrs) with COVID-19 were obtained from the Shenzhen Third People’s Hospital. Networks were constructed among 16 physical symptoms to describe relationships among symptoms. Subgroup network analyses were performed based on latent class analysis. We also conducted further analysis of the bridge symptoms among the symptom clusters.

**Results:**

Fever (rs=4.37) had the highest strength value, followed by chills (rs=4.20) and muscle aches (rs=3.91). Based on the values of bridge strength centrality, dizziness (rbs=0.79), runny nose (rbs=0.71), and fatigue (rbs=0.62) were identified as bridge symptoms. Among the three subgroups of symptom networks, cough had the highest strength values in the high and low symptom severity groups (rs=3.93 and 2.82), while fever had the highest strength value in the moderate symptom severity group (rs=3.50).

**Conclusion:**

We found that the core symptom was different in the high/low groups and moderate group, suggesting that personalized symptom management plans are essential to optimize the effectiveness of interventions. Keywords: COVID-19; Older adults; Physical symptom; Symptom network

